# Dietary *Artemisia ordosica* Krasch Supplementation Alters n-3 Polyunsaturated Fatty Acid Deposition and Lipid Metabolism in Cashmere Goat Meat

**DOI:** 10.3390/ani16131982

**Published:** 2026-06-26

**Authors:** Jintao Liu, Hao Yu, Shuhui Dong, Shangxiong Zhang, Zaccheaus Pazamilala Akonyani, Qingyue Zhang, Yongmei Guo, Xiaoyu Guo, Binlin Shi, Yanli Zhao, Sumei Yan

**Affiliations:** 1 Key Laboratory of Animal Nutrition and Feed Science of Inner Mongolia Autonomous Region, College of Animal Science, Inner Mongolia Agricultural University, Hohhot 010018, China; jintaoliu@emails.imau.edu.cn (J.L.); haoyu@emails.imau.edu.cn (H.Y.); shuhuidong@emails.imau.edu.cn (S.D.); zakonyani@emails.imau.edu.cn (Z.P.A.); zhangqy2024@imau.edu.cn (Q.Z.); ymguo2015@163.com (Y.G.); gxy2024@imau.edu.cn (X.G.); shibl@imau.edu.cn (B.S.); 2 National Technology Innovation Center for Prataculture, Hohhot 010010, China; ggsw@mengcao.com

**Keywords:** *Artemisia ordosica* Krasch, n-3 polyunsaturated fatty acids, antioxidant capacity, multi-omics, ruminal biohydrogenation, AMPK signaling pathway

## Abstract

Enriching meat with n-3 polyunsaturated fatty acids (n-3 PUFAs) is of considerable nutritional interest because of the well-documented cardioprotective and anti-inflammatory properties of these fatty acids in the human diet. This study investigated the potential effects of dietary *Artemisia ordosica* Krasch (ARI) supplementation on muscle n-3 PUFA deposition in Albas White Cashmere goats, possibly mediated through the regulation of antioxidant capacity, lipid oxidation, and metabolism. Under the present experimental conditions, ARI supplementation did not significantly affect growth performance, but significantly reduced ruminal C18:0 content (*p* < 0.05) and increased n-3 PUFA levels (C18:3n3, C22:6n3) in rumen fluid, plasma, liver, and *Longissimus dorsi*, and the consequent elevation of the muscle PUFA-to-saturated fatty acid ratio (P/S; *p* < 0.05). Concurrently, ARI supplementation enhanced ruminal and systemic antioxidant capacity, as evidenced by increased glutathione peroxidase (GPx) and superoxide dismutase (SOD) activities, elevated total antioxidant capacity (T-AOC), and upregulated expression of antioxidant-related genes (*GSR*, *GOR*, *SOD2*). Integrated multi-omics analyses revealed that these improvements may be associated with the reduced relative abundance of the core biohydrogenating bacterium *s_Butyrivibrio_fibrisolvens* and de novo saturated fatty acid synthesis, and potential involvement of the AMPK signaling pathway, with downregulation of *FASN*; coordinated upregulation of *CD36*, *ACSL3*/*4*, and *ELOVL6*/*7*; and upregulation of *PPARGC1A*, collectively suggesting a promotion of FA oxidation and n-3 PUFA deposition. Collectively, these findings suggest that ARI-promoted n-3 PUFA enrichment in goat meat may be associated with the coordinated modulation of ruminal biohydrogenation, systemic antioxidant capacity, and intermediary lipid metabolism, providing a potential nutritional strategy for developing functional cashmere goat meat.

## Simple Summary

Consumers increasingly seek healthier meat, yet intensive barn-feeding systems often reduce the levels of beneficial n-3 polyunsaturated fatty acids (n-3 PUFAs) and increase saturated fats in cashmere goat meat. This study tested whether adding *Artemisia ordosica* Krasch, a native Chinese plant, to the diet of cashmere goats could improve the meat’s nutritional quality. The goats that received the plant supplement showed markedly higher levels of healthy n-3 PUFAs (including α-linolenic acid and DHA) and lower saturated fat in their muscle, without affecting growth. These improvements were associated with a reduced abundance of certain ruminal microorganisms that convert unsaturated fatty acids which are more beneficial to human health into saturated fatty acids, enhanced antioxidant defenses, and the activation of metabolic pathways potentially involved in n-3 PUFA deposition. These findings suggest that *Artemisia ordosica* Krasch may serve as a natural feed ingredient to produce healthier cashmere goat meat rich in n-3 PUFAs, offering benefits for both livestock producers and health-conscious consumers.

## 1. Introduction

The profile of fatty acids (FAs) in meat plays a central role in defining its nutritional value and subsequent effects on human health [1]. Excessive intake of diets rich in saturated fatty acids (SFAs) has long been consistently associated with a higher incidence of cardiovascular diseases and metabolic syndrome. Some studies have reported that stearic acid (C18:0) is positively correlated with an increased risk of chronic diseases [2]. By contrast, polyunsaturated FAs (PUFAs), particularly the n-3 family members such as α-linolenic acid (C18:3n3) and docosahexaenoic acid (C22:6n3), confer well-documented health advantages, ranging from a reduction in atherosclerotic lesions to the optimization of serum lipid profiles [3,4]. However, when the dietary ratio of n-6 to n-3 PUFAs becomes excessively high, it can competitively suppress the activity of enzymes responsible for n-3 PUFA metabolism, thereby limiting their protective effects and fostering the development of chronic cardiometabolic diseases [4]. Hence, formulating nutritional regimens that boost n-3 PUFA consumption, lower SFA levels, and sustain a low n-6/n-3 ratio is fundamental to advancing public health via dietary means.

With rising health consciousness, consumers increasingly seek out premium meat products, including goat meat. The Albas White Cashmere goat, a native, dual-purpose breed from the grasslands of northwestern China, has long been valued under pastoral grazing systems for its succulent texture and palatable flavor [5]. Yet, due to the combined pressures of seasonal feed scarcity, limited grazing land, and progressive grassland deterioration [6], the industry is steadily converting to intensive barn-feeding systems [6]. This shift in production strategy frequently leads to unfavorable modifications in chevon’s FA profile: SFA concentrations (especially C18:0) increase, the n-6/n-3 ratio rises, and n-3 PUFA content declines, all of which impair the nutritional and health-enhancing qualities of the chevon [7]. Fortunately, incorporating plants or their extracts that are abundant in flavonoids and polyphenols into the goats’ diet has been demonstrated to offset these detrimental shifts, reducing SFA content and the n-6/n-3 ratio while simultaneously elevating n-3 PUFA proportions in ruminant muscle [8,9]. Consequently, refining the FA profile of cashmere goat meat through precisely targeted nutritional interventions has become a key focus of current research.

*Artemisia ordosica* Krasch (*ARI*) is a broadly distributed Chinese medicinal herb in the semi-desert regions of western and northern China, and is rich in bioactive flavonoids, polyphenols, and polysaccharides [10]. Our previous study found that the water-extracted lyophilized powder of ARI contains 29.43% polysaccharides [11]. Further investigations demonstrated that these extracts improve antioxidant capacity in cashmere goats [12], and these polysaccharides also exhibit potential for modulating lipid metabolism and reshaping the FA profile of animal-derived foods [13]. However, research on the role of ARI in modulating lipid metabolism and FA profile in meat goats remains limited, and its underlying mechanisms are not yet fully elucidated. The formation of the muscle FA profile is orchestrated by a complex network involving ruminal biohydrogenation, systemic FA oxidation, elongation, and desaturation [14], all of which can be influenced by the host’s oxidative status. Metabolomics enables the delineation of tissue-specific shifts in core lipid metabolites and associated pathways, while transcriptomics reveals differentially expressed genes governing FA metabolism and deposition. The integration of these multi-omics dimensions provides a powerful approach to systematically elucidate causal mechanisms beyond single-level observations [15].

We hypothesized that dietary ARI supplementation could modulate the muscle FA profile of Albas White Cashmere goats by regulating lipid metabolism and enhancing antioxidant defense, thereby reducing C18:0 content, promoting n-3 PUFA deposition, and improving the nutritional value of the cashmere goat meat. This study evaluated the effects of ARI on n-3 PUFA deposition in cashmere goat meat and elucidates the underlying regulatory mechanisms using an integrated multi-omics approach.

## 2. Materials and Methods

### 2.1. Animals, Experimental Design, and Diets

Forty healthy, castrated male Albas White Cashmere goats (4 months old, initial BW 18.0 ± 0.5 kg) were assigned to a completely randomized design. These animals were evenly and randomly assigned to two dietary treatment groups (*n* = 20 per group): (i) a control group (CON) receiving a basal diet, and (ii) an ARI group, in which 3% ARI replaced an equivalent portion of the mixed roughage in the basal ration. The ARI was collected in August from Ordos City, Inner Mongolia, China (110.03° E, 40.41° N), then naturally shade-dried prior to incorporation into the feeds.

The trial was undertaken at the research facilities of Inner Mongolia Agricultural University, Hohhot, China, spanning 104 days total: a 14-day adaptation phase immediately followed by a 90-day formal feeding period. The formal trial was subsequently subdivided into three 30-day intervals (early, middle, and late). Individual pens with concrete floors housed each goat, with free access to fresh water throughout the experiment. The diets satisfied the nutrient demands of growing cashmere goats (NY/T 816-2021) [16]. Table 1 details the ingredient and chemical composition of the test diets, while the dietary FA profile is presented in Appendix A Table A1. The dietary nutrient composition and fatty acid profile were largely comparable between the two groups. A total mixed ration (TMR) was fed twice daily at 07:00 and 18:00 h.

### 2.2. Sample Collection

#### 2.2.1. Feed Intake and Growth Performance

Daily records were kept for feed offered and feed refusals to compute dry matter intake (DMI). Body weight was recorded at the beginning of the formal feeding period and at the end of the trial (day 90), enabling the calculation of average daily gain (ADG) and feed-to-gain ratio (F/G).

#### 2.2.2. Diet Samples

Representative diet samples were taken during each of the three experimental phases (day 30, day 60, day 90) for subsequent analysis of FA profiles.

#### 2.2.3. Blood Samples

At the conclusion of the feeding trial (day 90), prior to slaughter, fasting blood specimens were drawn from the jugular vein into vacuum tubes devoid of anticoagulant. After centrifugation at 3500× *g* for 15 min at 4 °C, the separated serum was aliquoted and preserved at −20 °C (for biochemical and antioxidant assays) or at −80 °C for subsequent metabolomic evaluation.

#### 2.2.4. Tissue Samples

Immediately following slaughter, *Longissimus dorsi* specimens (excised from the 12th–13th rib interspace) as well as liver specimens were harvested. Aliquots designated for FA analysis, enzyme activity assays, antioxidant measurements, transcriptomics, and metabolomics were snap-frozen in liquid N_2_ and maintained at −80 °C pending analysis.

#### 2.2.5. Rumen Fluid Samples

Rumen fluid was collected right after slaughter, then filtered through quadruple-layered cheesecloth to remove feed debris. The strained fluid was kept at −80 °C for metagenomic and metabolomic assessments and antioxidant enzyme assays.

### 2.3. Growth Performance Calculations

Average daily gain (ADG, kg/d) was derived as (final BW − initial BW)/number of days. Dry matter intake (DMI, kg/d) was computed as total dry matter consumed divided by the number of days. Feed-to-gain ratio (F/G) was determined as total DMI (kg)/total weight gain (kg).

### 2.4. FA Profile Analysis

FA profiles were determined in the diet, rumen fluid, plasma, liver, and *Longissimus dorsi* of the same six goats per group (*n* = 6). FA methyl esters (FAMEs) were derivatized from lyophilized samples (tissues, feed, rumen fluid, and plasma) following a modified two-step procedure originally described by O’Fallon et al. [17]. In brief, samples were saponified with 10 M KOH in methanol at 55 °C for 1.5 h, then methylated with concentrated H_2_SO_4_ at the same temperature for an additional 1.5 h. FAMEs were extracted using n-hexane and subsequently separated on an Agilent 7890B GC (Agilent, Santa Clara, CA, USA) fitted with a flame ionization detector and an SP-2560 capillary column (100 m × 0.25 mm × 0.20 μm; Supelco, Bellefonte, PA, USA). The temperature program, injector settings, and detector parameters were as detailed in [18]. Individual FAMEs were identified and quantified by comparison with a 37-component FAME standard mixture (Sigma-Aldrich, St. Louis, MO, USA). The results are expressed as a percentage of total identified FAs.

### 2.5. Determination of Lipid Metabolism-Related Enzyme and Transporter Protein Abundances

ELISA kits (R&D Systems, Minneapolis, MN, USA) were used to determine the protein abundances of fatty acid synthase (FAS, Cat. No. FG231), acetyl-CoA carboxylase (ACC, Cat. No. FG219), elongation of very long-chain fatty acids protein 5 (ELOVL5, Cat. No. FG203), hormone-sensitive lipase (HSL, Cat. No. FG239), stearoyl-CoA desaturase (SCD, Cat. No. FG217), and the fatty acid translocase CD36 (Cat. No. FG225) in the serum, liver, and *Longissimus dorsi* from the same six goats per group used for the FA analysis (*n* = 6), strictly adhering to the manufacturers’ instructions. Tissue specimens were homogenized in ice-cold phosphate-buffered saline (PBS, pH 7.4), then centrifuged at 12,000 × *g* for 10 min at 4 °C to obtain the supernatant. The total protein concentration of each tissue homogenate was measured via the bicinchoninic acid (BCA) method [18], and the ELISA-derived protein concentrations were normalized to the total protein content of each sample. The results are expressed as ng/mg protein.

### 2.6. Serum Biochemical Markers

To measure the serum concentrations of albumin (ALB), alkaline phosphatase (ALP), aspartate aminotransferase (AST), β-hydroxybutyrate (BHB), blood urea nitrogen (BUN), glucose (GLU), high-density lipoprotein cholesterol (HDL-C), low-density lipoprotein cholesterol (LDL-C), non-esterified fatty acids (NEFA), total bilirubin (TBIL), total cholesterol (CHO), and triglycerides (TG), a Hitachi 7020 auto-analyzer (Hitachi High-Tech Corporation, Tokyo, Japan) [19] and Wako commercial kits from MedicalSystem Biotechnology Co., Ltd. (Ningbo, China) were used.

### 2.7. Antioxidant Enzyme Capabilities

Commercial assay kits from the Nanjing Jiancheng Bioengineering Institute (Nanjing, China) were employed to assess the levels of total antioxidant capacity (T-AOC, Cat. No. A015-1-2), glutathione peroxidase (GPx, Cat. No. A005-1-2), superoxide dismutase (SOD, Cat. No. A001-3-2), and malondialdehyde (MDA, Cat. No. A003-1-2) in the serum, rumen fluid, liver, and Longissimus dorsi of six goats per group (*n* = 6). Tissue specimens were homogenized in ice-cold saline at a ratio of 1:9 (weight to volume). Subsequently, the homogenates were centrifuged at 2500× *g* for 10 min at 4 °C to obtain the supernatant. All assays followed the manufacturer’s protocols. Protein levels were quantified by the BCA assay for data normalization [18].

### 2.8. Rumen Metagenomic Analysis

#### 2.8.1. DNA Extraction and Sequencing

Upon trial completion, rumen fluid samples were obtained from three goats chosen at random per group (*n* = 3), which were a subset of the six animals used for the targeted physiological assays. The samples were subjected to metagenomic sequencing. Genomic DNA was isolated using the E.Z.N.A.^®^ Soil DNA Kit (Omega Bio-tek, Norcross, GA, USA). A NanoDrop 2000 spectrophotometer (Thermo Scientific, Wilmington, DE, USA) assessed DNA concentration and purity, while agarose gel electrophoresis verified its integrity [20]. Libraries were prepared using the NEXTFLEX Rapid DNA-Seq Kit (Bioo Scientific, Austin, TX, USA) and then sequenced on an Illumina NovaSeq 6000 platform (Illumina, San Diego, CA, USA) (2 × 150 bp paired-end) at Majorbio Bio-pharm Technology Co., Ltd. (Shanghai, China).

#### 2.8.2. Bioinformatics Analysis

Raw sequencing reads underwent quality trimming via FastQC (v0.11.9; Babraham Bioinformatics, Cambridge, UK) and Sickle (v1.33) to remove adapters and low-quality sequences (quality score < 20, read length < 50 bp). Reads of host origin were eliminated by aligning to the goat reference genome (ARS1) with Bowtie2 (v2.4.2; Johns Hopkins University, Baltimore, MD, USA). Metagenomic assembly was performed using MEGAHIT (v1.2.9), retaining contigs longer than 300 bp. Taxonomic classification was carried out with the MetaOthello classifier against an integrated microbial genome database (NCBI RefSeq and GenBank). Differential species abundance (expressed as reads per kilobase per million, RPKM) was evaluated using the Wilcoxon rank-sum test, and biomarkers were identified by linear discriminant analysis effect size (LEfSe; LDA score > 2.0, *p* < 0.05). For functional annotation, genes were predicted from contigs using Prodigal (v2.6.3) and aligned to the Kyoto Encyclopedia of Genes and Genomes (KEGG) database (release 98.0) with DIAMOND (v0.9.24; Max Planck Institute for Informatics, Saarbrücken, Germany; e-value < 1 × 10^−5^). Differential abundance of KEGG Orthology (KO) was identified by the Wilcoxon rank-sum test with false discovery rate (FDR) adjustment (*p* < 0.05), followed by pathway enrichment analysis using Goatools (v0.6.5) and custom R scripts developed in R version 4.4.2 [21].

### 2.9. Untargeted Metabolomics

#### 2.9.1. Preparation of Biological Samples

Serum, rumen fluid, and liver specimens from the same six goats per group selected for the targeted analyses (*n* = 6) were analyzed by an untargeted metabolomics approach [22]. In brief, aliquots (100 μL for liquids, 50 mg for tissues) were mixed with a methanol–water solution (4:1, *v*/*v*) spiked with an internal standard (2-chlorophenylalanine), vortexed, incubated at −20 °C for 30 min, and subsequently centrifuged. The clear upper phase was then dried under nitrogen and reconstituted in acetonitrile–water (1:1, *v*/*v*) prior to injection. A pooled quality control (QC) sample was injected periodically to monitor system stability [23].

#### 2.9.2. LC-MS Analysis

A Vanquish Horizon UHPLC system coupled with a Q Exactive HF-X mass spectrometer (Thermo Scientific, Waltham, MA, USA) was employed for metabolite profiling. An ACQUITY UPLC HSS T3 column (2.1 × 100 mm, 1.8 μm; Waters, Milford, MA, USA) was utilized to achieve chromatographic separation. Data were collected under both positive and negative ion detection modes.

#### 2.9.3. Computational Analysis of Metabolomic Data

Raw LC-MS data were first processed with Progenesis QI (v2.3; Nonlinear Dynamics, Newcastle, UK) for peak detection, alignment, and normalization [24]. Metabolites were subsequently identified by matching their exact masses (mass tolerance < 10 ppm) and MS/MS fragmentation spectra against the Human metabolome database (HMDB) (http://www.hmdb.ca/) and Metlin database (https://metlin.scripps.edu/) [25]. Multivariate statistical analyses, including partial least squares discriminant analysis (PLS-DA) and orthogonal partial least squares discriminant analysis (OPLS-DA), were performed using the ropls package (v1.22.0) in R. Differential metabolites were screened based on the following combined criteria: variable importance in projection (VIP) > 1.0 derived from the OPLS-DA model, absolute log_2_ fold change (|log_2_FC|) ≥ 1, and a statistically significant *p* < 0.05 (Student’s *t*-test). Finally, the identified differential metabolites were subjected to Kyoto Encyclopedia of Genes and Genomes (KEGG) pathway enrichment analysis on the Omicshare platform (Majorbio, Shanghai, China, https://cloud.majorbio.com). The significance of enrichment was evaluated by hypergeometric test, followed by Benjamini–Hochberg (BH) multiple testing correction. Pathways with a false discovery rate (FDR)-adjusted *Q* < 0.05 were considered significantly enriched.

### 2.10. Transcriptome Analysis of Longissimus Dorsi

#### 2.10.1. RNA Extraction and Sequencing

*Longissimus dorsi* samples from the same goats used for metagenomic analysis (*n* = 3) were subjected to transcriptomic profiling. TRIzol reagent (Invitrogen, Carlsbad, CA, USA) was used for total RNA extraction. RNA concentration, purity, and integrity (RIN > 8.0) were assessed using a NanoDrop 2000 and an Agilent 2100 Bioanalyzer (Agilent Technologies, Santa Clara, CA, USA). Libraries were generated from strand-specific RNA using the TruSeq Stranded mRNA Library Prep kit (Illumina, San Diego, CA, USA) [26] and sequenced on an Illumina HiSeq X Ten platform (2 × 150 bp paired-end reads) at Majorbio Bio-pharm Technology Co., Ltd. (Shanghai, China).

#### 2.10.2. Bioinformatics Analysis

Raw reads were trimmed with SeqPrep and Sickle to remove adapters and low-quality bases. HISAT2 (v2.1.0) was employed to align clean reads against the goat reference genome (ARS1) [27], and gene expression levels were quantified as transcripts per million (TPM) with RSEM (v1.3.1) [28]. Differential expression analysis was performed using DESeq2 (v1.30.1) in R; differentially expressed genes (DEGs) were defined as those with |log_2_ fold change| ≥ 1 and FDR-adjusted *p* < 0.05. Gene Ontology (GO) and KEGG pathway enrichment analyses were performed using Goatools (v0.6.5) and custom R scripts [21]. An FDR-adjusted *p*-value below 0.05 was taken as significant.

### 2.11. Validation by Quantitative Real-Time PCR (qRT-PCR)

To confirm the RNA-seq findings, the expression levels of nine genes related to lipid metabolism (*SCP2*, *FASN*, *ELOVL6*, *PRKAA1*, *CD36*, *ACSL4*, *ACSS3*, *PPARGC1A*, and *IGF1*) were measured by qRT-PCR in the *Longissimus dorsi* of the same six goats per group used for FA and antioxidant analyses (*n* = 6). First-strand cDNA was synthesized from 1 μg total RNA using the PrimeScript™ RT Reagent Kit with gDNA Eraser (Takara, Dalian, China). SYBR^®^ Premix Ex Taq™ II (Takara) was used for qRT-PCR on an ABI 7500 Real-Time PCR System (Applied Biosystems, Foster City, CA, USA). Gene expression levels were derived via the 2^−ΔΔCt^ method [29] with Beta-2-microglobulin (*B2M*) serving as the reference gene. All primer pairs, including those for *B2M* and the target genes, exhibited acceptable amplification efficiencies ranging from 92% to 102%, with correlation coefficients (R^2^) > 0.99. Melt curve analysis showed single, sharp peaks, confirming the specificity of amplification. These parameters meet the acceptance criteria recommended by the MIQE guidelines [30]. The primer sequences, amplicon sizes, and annealing temperatures for all the genes are listed in Appendix A Table A2.

### 2.12. Statistical Analysis

The normality of the data was first assessed using the Shapiro–Wilk test [31]. For comparisons between two groups, Welch’s *t*-test (which does not assume equal variances) was applied to evaluate differences in the FA profiles (ruminal fluid, plasma, liver, and muscle), enzyme activities (serum, muscle, and liver), and blood biochemical indices. The data are presented as means ± standard error of the mean (SEM). All statistical analyses were performed using SAS 9.2 software (SAS Institute Inc., Cary, NC, USA). A significance threshold of *p* < 0.05 was adopted.

## 3. Results

### 3.1. Effect of ARI Supplementation on Growth Performance

Under the present experimental conditions, dietary ARI supplementation did not significantly affect ADG, DMI, or FGR (*p* > 0.05; Table 2). However, it is noteworthy that ADG was numerically lower and FGR was numerically higher in the ARI group.

### 3.2. Effect of ARI Supplementation on FA Profile

#### 3.2.1. Rumen Fluid FA Profile

Table 3 reveals that ARI supplementation markedly modified the FA profile of rumen fluid. The ARI group exhibited lower (*p* < 0.05) concentrations of C13:0, C16:0, C18:0, total SFA, n-6 LCPUFA, and the n-6/n-3 ratio. Conversely, higher (*p* < 0.05) concentrations of C18:1n9c, C18:1n9t, C18:3n3, C20:3n3, total unsaturated fatty acids (UFA), n-3 PUFA, and the UFA/SFA (U/S) and PUFA/SFA (P/S) ratios were observed in the ARI group.

#### 3.2.2. Plasma FA Profile

In plasma (Table 3), ARI supplementation significantly reduced (*p* < 0.05) C18:0 and the n-6/n-3 ratio, while increasing (*p* < 0.05) the concentrations of C21:0, C22:0, C16:1, C17:1, C18:1n9t, C18:2n6t, C20:2n6, C20:3n6, C18:3n3, C20:3n3, C20:5n3, C22:6n3, C22:2n6, total PUFA, n-3 LCPUFA, n-3 PUFA, and the P/S ratio.

#### 3.2.3. Liver FA Profile

In the liver (Table 4), ARI supplementation decreased (*p* < 0.05) the concentrations of C18:0, C20:0, C22:0, C20:3n6, and total SFA. Concurrently, it increased (*p* < 0.05) the concentrations of C18:1n9c, C18:3n3, C22:6n3, total UFA, MUFA, n-3 PUFA, n-3 LCPUFA, and the U/S and P/S ratios.

#### 3.2.4. Longissimus Dorsi FA Profile

Lower concentrations (*p* < 0.05) of C14:0, C18:0, C20:0, C20:1, total SFA, n-6 PUFA, and the n-6/n-3 ratio were observed in the *Longissimus dorsi* of ARI-fed goats (Table 4), alongside higher (*p* < 0.05) concentrations of C18:1n9t, C18:3n3, C22:6n3, total UFA, n-3 PUFA, n-3 LCPUFA, and the U/S and P/S ratios.

### 3.3. Effect of Lipid Metabolism-Related Proteins/Enzymes

**Figure 1:**
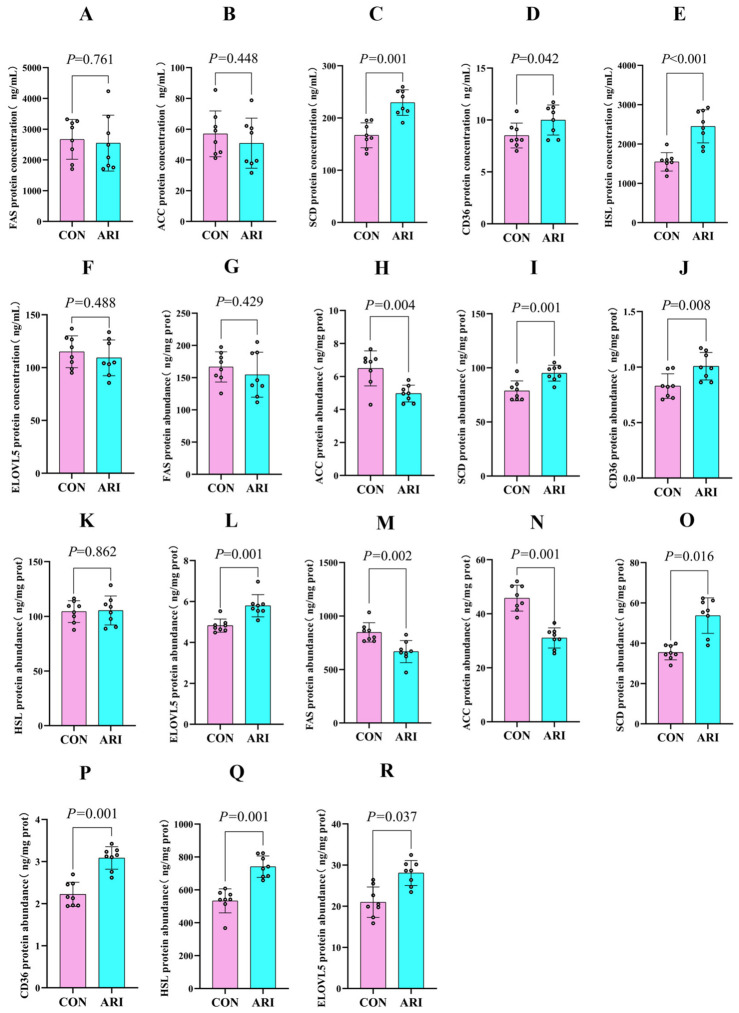
The effects of ARI on lipid metabolism-related proteins/enzymes in the serum, liver, and *Longissimus dorsi* samples. CON = Control diet; ARI *= Artemisia ordosica* Krasch diet. In the bar plot, each dot represents the measured value of an individual sample within the group. (**A**–**F**) depict serum lipolytic enzyme protein abundance; (**G**–**L**) show hepatic protein abundance; (**M**–**R**) present protein abundance in the *Longissimus dorsi*.

ARI supplementation significantly modulated the protein abundance of key lipid metabolism enzymes (Figure 1). The ARI group exhibited elevated protein abundance of SCD in the serum (Figure 1C; *p* = 0.001), liver (Figure 1I; *p* = 0.001), and *Longissimus dorsi* (Figure 1O; *p* = 0.016) samples. CD36 protein abundance was also higher in the ARI group across all three tissues (serum: Figure 1D, *p* = 0.042; liver: Figure 1J, *p* = 0.008; muscle: Figure 1P, *p* = 0.001). HSL protein abundance was significantly increased in the serum (Figure 1E; *p* < 0.001) and muscle (Figure 1Q; *p* = 0.001) of the ARI group.

Conversely, ACC protein abundance was significantly lower in the liver of the ARI group (Figure 1H; *p* = 0.004). In the *Longissimus dorsi*, the protein abundance of FAS (Figure 1M; *p* = 0.002) and ACC (Figure 1N; *p* = 0.001) were significantly reduced, while ELOVL5 protein abundance was significantly higher in the ARI group (liver: Figure 1L, *p* = 0.001; muscle: Figure 1R, *p* = 0.037).

### 3.4. Effect of ARI Supplementation on Serum Biochemical Indices

**Figure 2:**
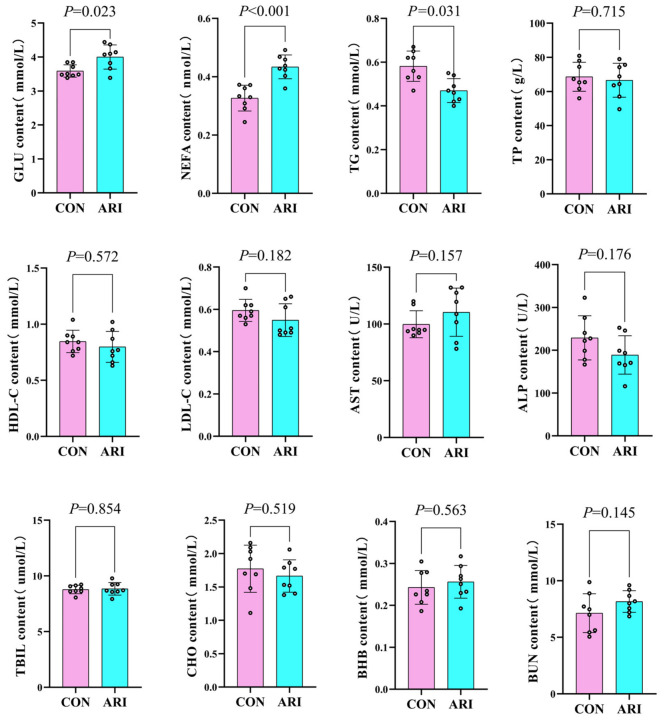
The effects of ARI on the blood biochemical indices of cashmere goats. CON = Control diet, and ARI = *Artemisia ordosica* Krasch diet. In the bar plot, each dot represents the measured value of an individual sample within the group.

Figure 2 reveals that ARI supplementation elevated serum GLU (*p* = 0.023) and NEFA (*p* < 0.001) levels, while reducing TG (*p* = 0.031), relative to the CON group. The remaining serum biochemical parameters showed no significant changes (*p* > 0.05).

### 3.5. Effect of ARI Supplementation on Antioxidant Enzyme Activities

**Figure 3:**
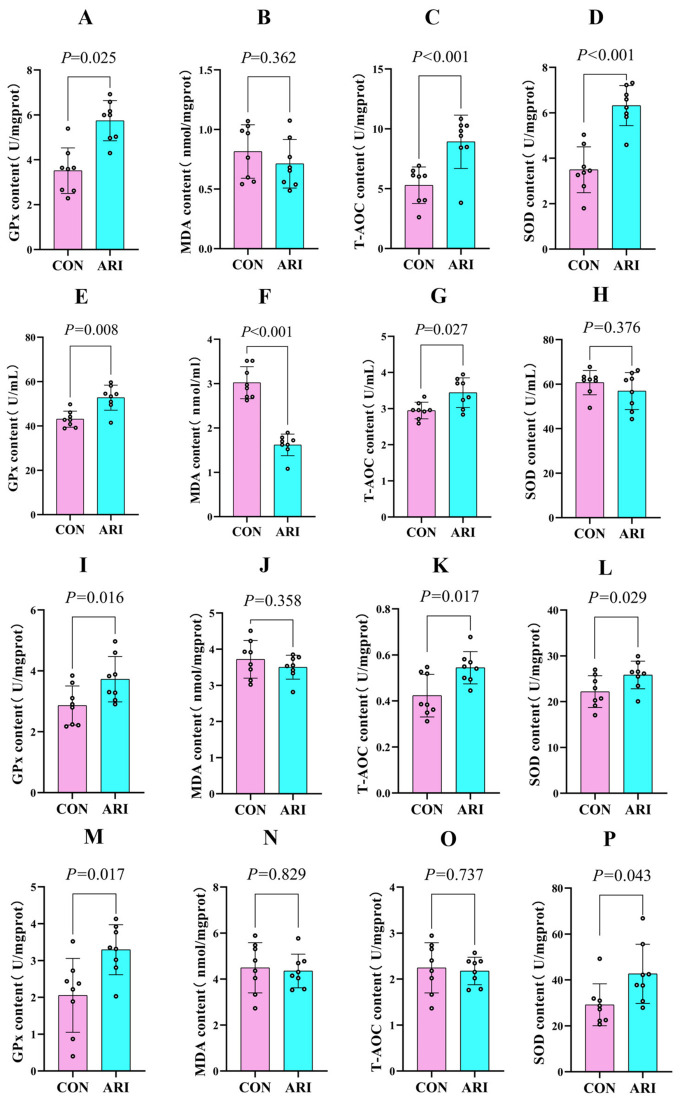
The effects of ARI on antioxidant enzyme activities in ruminal fluid, serum, liver, and *Longissimus dorsi*. CON = Control diet, and *ARI* = *Artemisia ordosica* Krasch diet. In the bar plot, each dot represents the measured value of an individual sample within the group. (**A**–**D**), (**E**–**H**), (**I**–**L**) and (**M**–**P**) represent the antioxidant enzyme activities in the ruminal fluid, serum, liver, and *Longissimus dorsi*, respectively.

ARI supplementation enhanced antioxidant capacity across multiple tissues (Figure 3). Compared with the CON group, the ARI group showed significantly increased GPx activity in the rumen fluid (Figure 3A; *p* = 0.025), serum (Figure 3E; *p* = 0.008), liver (Figure 3I; *p* = 0.016), and muscle (Figure 3M; *p* = 0.017) samples. T-AOC was elevated in the rumen fluid (Figure 3C; *p* < 0.001), serum (Figure 3G; *p* = 0.027), and liver (Figure 3K; *p* = 0.017) samples. SOD activity was higher in the rumen fluid (Figure 3D; *p* < 0.001), liver (Figure 3L; *p* = 0.029), and muscle (Figure 3P; *p* = 0.043) samples. Conversely, MDA concentration was significantly reduced in the serum of the ARI group (Figure 3F; *p* < 0.001).

### 3.6. Effect of ARI Supplementation on Rumen Metagenomic Profiles

#### 3.6.1. Ruminal Microbial Diversity and Composition

**Figure 4:**
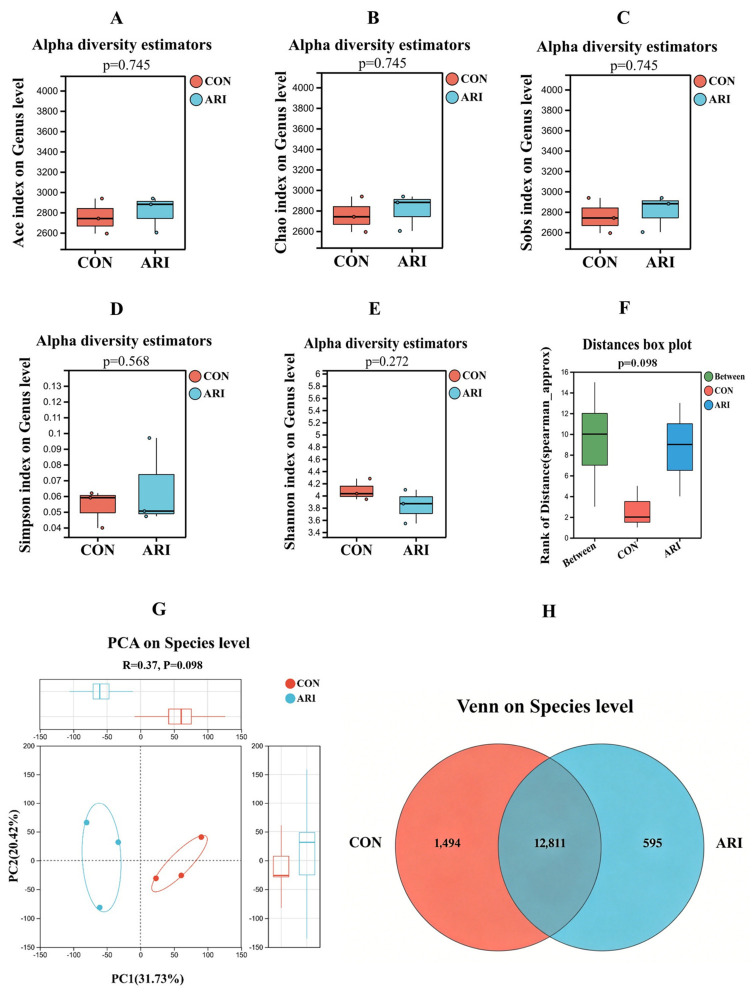
Analysis of rumen microbiota alpha (α)- and beta (β)-diversity with species and functional composition profiling. CON = Control diet, and ARI *= Artemisia ordosica* Krasch diet. (**A**–**E**) Rumen microbiota α-diversity (ARI vs. CON). (**F**) ANOSIM of microbial β-diversity. (**G**) Principal component analysis (PCA) of inter-sample diversity for rumen fluid microbiota at the species level. (**H**) A Venn diagram illustrating interspecies compositional differences in rumen fluid between the two groups.

While alpha-diversity indices (Figure 4A–E) and overall beta-diversity (Figure 4F) at the genus level were not significantly altered by ARI, PCA at the species level revealed a clear separation between the CON and ARI groups (Figure 4G). Venn diagram analysis identified 1494 and 595 unique species in the CON and ARI groups, respectively (Figure 4H), indicating a significant reshaping of the species-level community.

#### 3.6.2. Differential Species and Functional Biomarkers

**Figure 5:**
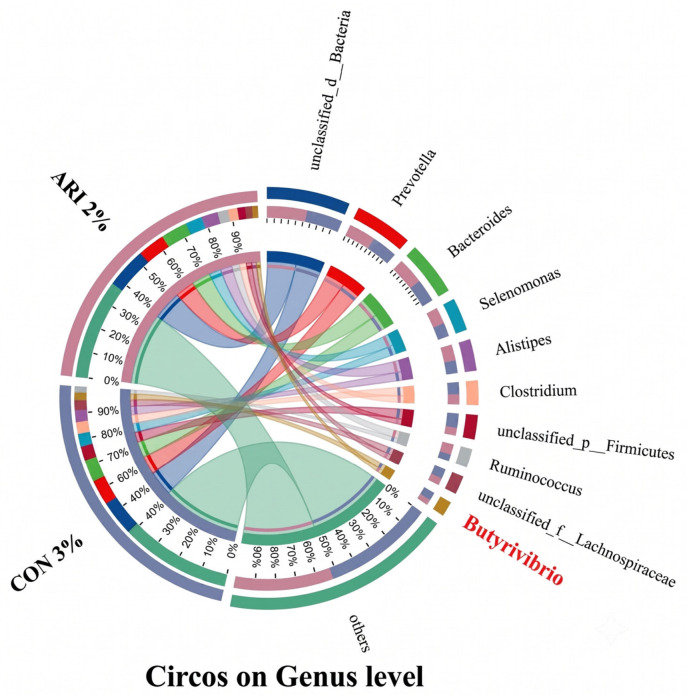
Analysis of genus-level sample-species (function) Circos diagrams. CON = Control diet; ARI = *Artemisia ordosica* Krasch diet. Presents a Circos plot to visualize the distribution of dominant bacterial genera across sample groups. On the left side, the first (outer) layer represents the sample groups, and the second (inner) layer uses colored ribbons to link each group to the taxa on the right, indicating distribution. On the right side, the first (outer) layer displays the dominant genera (color-coded by taxonomy), while the second (inner) layer illustrates, via split-colored ribbon segments, the proportional abundance contribution of each genus to the two connected groups. The *Butyrivibrio* genus, highlighted in red, represents a key ruminal biohydrogenation bacterium. The percentage values following CON and ARI indicate its relative abundance within each respective group.

**Figure 6:**
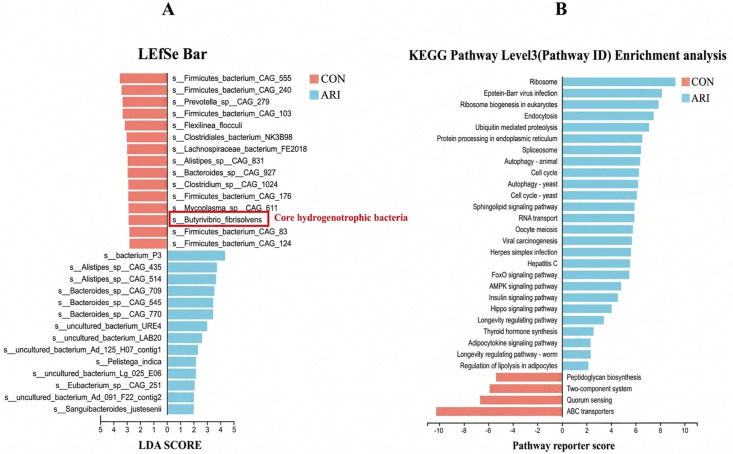
LefSe discriminant analysis of species and KEGG functional enrichment analysis in the Rumen Microbial Gene Catalog. CON = Control diet, and ARI = *Artemisia ordosica* Krasch diet. (**A**) shows the LDA bar plot derived from the LEfSe analysis, indicating the microbial taxa with significant discriminative power between the two groups and their corresponding LDA scores. Higher LDA scores signify a greater contribution of the respective taxon’s abundance to the observed intergroup differences. (**B**) KEGG functional enrichment analysis in the Rumen Microbial Gene Catalog. This analysis visualizes the top 30 significantly enriched functional pathways (absolute ReporterScore ≥ 1.65) by default. The horizontal axis represents the ReporterScore, while the vertical axis lists the identified metabolic pathways. The color of each bar indicates the experimental group in which the corresponding pathway was significantly enriched.

The relative abundance of *Butyrivibrio* was 2% in the ARI group and 3% in the CON group (Figure 5). LEfSe identified *s_Butyrivibrio_fibrisolvens* as a core biohydrogenation-related taxon with a high LDA score in the CON group (Figure 6A, Table 5). No single *Butyrivibrio*-related bacterium predominated in the ARI group.

#### 3.6.3. KEGG Functional Enrichment Analysis

**Figure 7:**
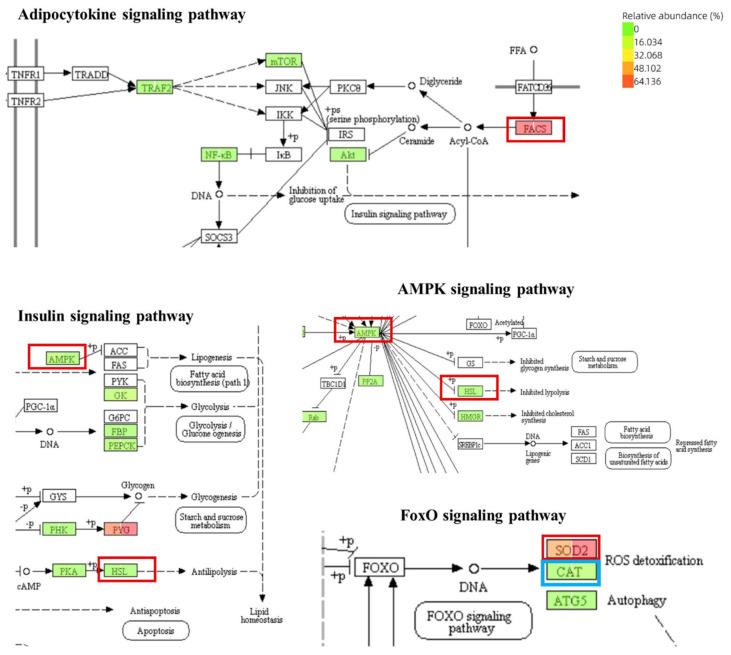
A visualization of KEGG-enriched pathways and key enzyme abundance profiling from the rumen microbial gene set. This heatmap visualizes variations in enzyme abundance among the sample groups, with color intensity scaled according to the color key. We have outlined boxes in red or blue to mark enzymes whose genes were significantly upregulated or downregulated in the ARI group, respectively.

KEGG enrichment analysis of the rumen metagenome revealed significantly enriched pathways related to lipid metabolism and cellular signaling, including the AMPK, insulin, adipocytokine, and FoxO signaling pathways in the ARI group (Figure 6B, Table 6). This enrichment was associated with increased relative abundance of key genes such as *PRKAA*/*AMPK*, *LIPE*/*HSL*, *SOD2*, and *GSR*/*GOR* (Figure 7).

### 3.7. Effect of ARI Supplementation on Metabolomic Profiles

#### 3.7.1. Multivariate Analysis

**Figure 8:**
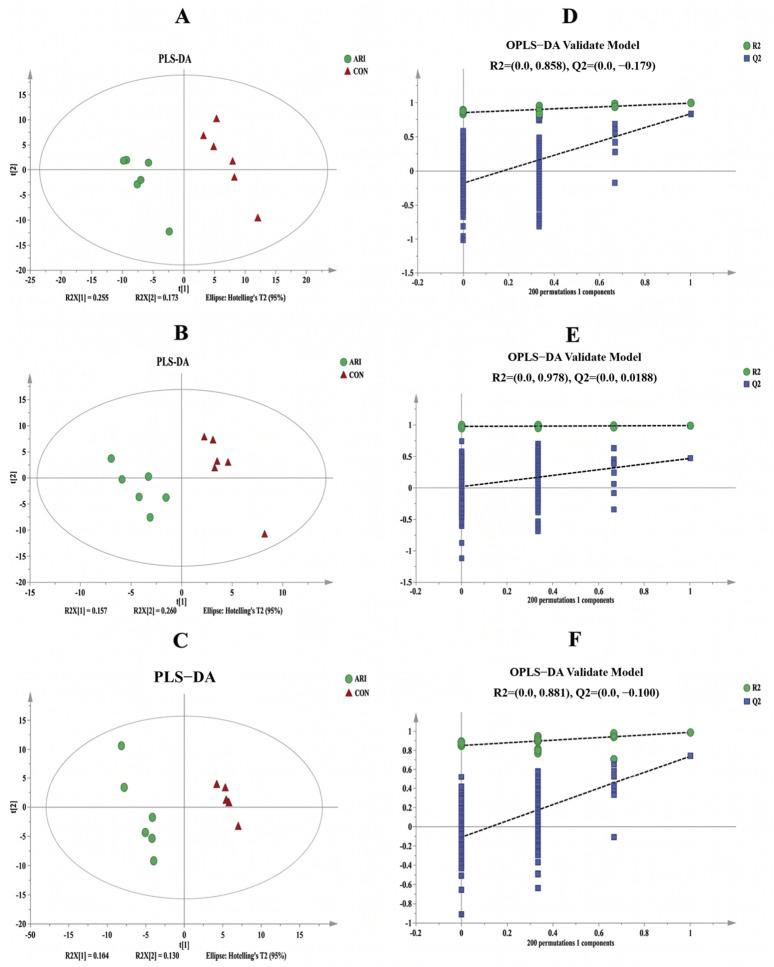
Score plots derived from PLS-DA and OPLS-DA models are displayed. CON = Control diet, and ARI = *Artemisia ordosica* Krasch diet. (**A**–**C**) show the PLS-DA score plots for the rumen fluid, serum, and liver samples, respectively. (**D**–**F**) display the orthogonal projections to latent structures-discriminant analysis (OPLS-DA) permutation test score plots for the corresponding samples. A permutation test Q^2^Y regression line intercept < 0.05 indicates that the model is robust, reliable, and not overfitted.

PLS-DA score plots demonstrated clear separation between the CON and ARI groups in ruminal fluid, serum, and liver metabolomes (Figure 8A–C). For datasets of rumen fluid metabolomes (Figure 8A,D), the PLS-DA model parameters were R^2^X = 0.428, R^2^Y = 0.858, and Q^2^ = −0.179. For datasets of serum metabolomes (Figure 8B,E), the model parameters were R^2^X = 0.417, R^2^Y = 0.978, and Q^2^ = 0.0188. For datasets of live metabolomes (Figure 8C,F), the model parameters were R^2^X = 0.284, R^2^Y = 0.881, and Q^2^ = −0.100. All three datasets and the permutation tests confirm that the models are free of overfitting. The consistently low or negative Q^2^ values demonstrate that the separations observed between the ARI and CON groups arise from true biological differences, thus validating the models for subsequent differential metabolite identification and biological interpretation.

#### 3.7.2. Differential Metabolite Identification

**Figure 9:**
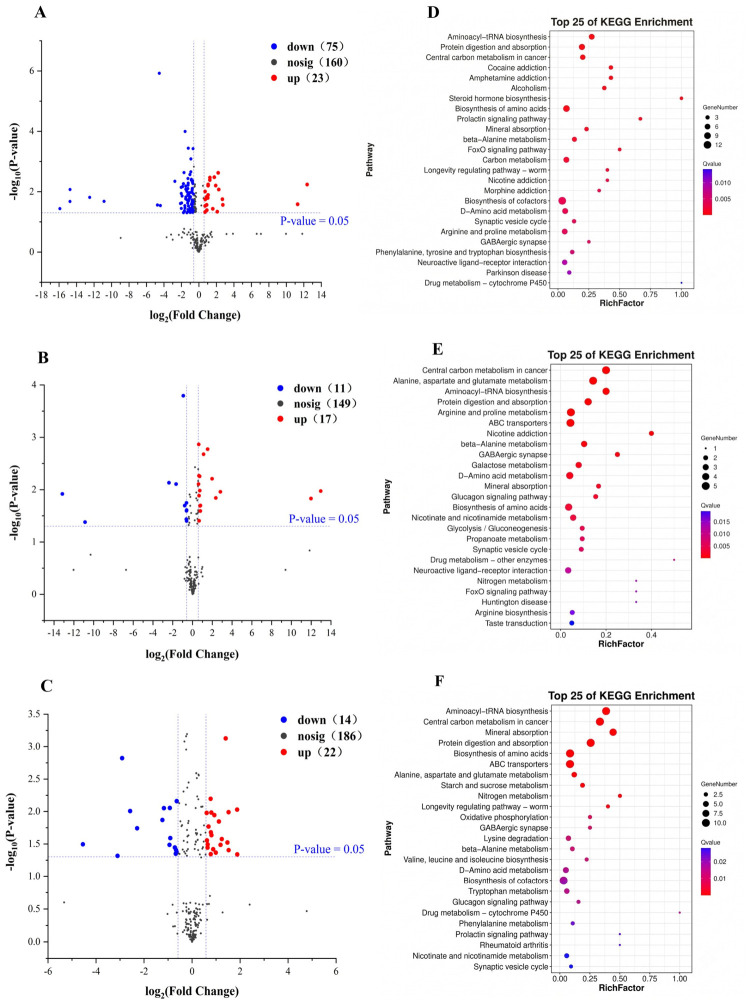
Volcano plots of differential metabolites and bubble plots of KEGG functional enrichment analysis of differential metabolites (ARI vs. CON). CON = Control diet, and ARI = *Artemisia ordosica* Krasch diet. ARI vs. CON: The ARI group compared to the CON group. (**A**–**C**) Volcano plots of differential metabolites in the rumen fluid, serum, and liver samples between the ARI and CON groups, respectively, with red, blue, and gray indicating upregulated, downregulated, and unchanged metabolites, respectively. (**D**–**F**) KEGG enrichment bubble plots of the differential metabolites in the rumen fluid, serum, and liver samples between the ARI and CON groups, respectively.

Volcano plot analysis identified 258, 177, and 222 differential metabolites in ruminal fluid, serum, and liver, respectively (Figure 9A–C).

#### 3.7.3. KEGG Pathway Enrichment of Differential Metabolites

ARI-responsive metabolites were significantly enriched in pathways related to amino acid and lipid metabolism in ruminal fluid, serum and liver, such as aminoacyl-tRNA biosynthesis, beta-alanine metabolism (Figure 9D–F, Table 7). The key ruminal metabolite beta-alanine was downregulated, while glycine was upregulated. In serum, the key metabolites glucose-1-phosphate and GABA were upregulated. In the liver, the key metabolites glutamine and GABA were upregulated (Table 7).

### 3.8. Transcriptomic Responses of Longissimus Dorsi to ARI Supplementation

#### 3.8.1. Differential Gene Expression Analysis

**Figure 10:**
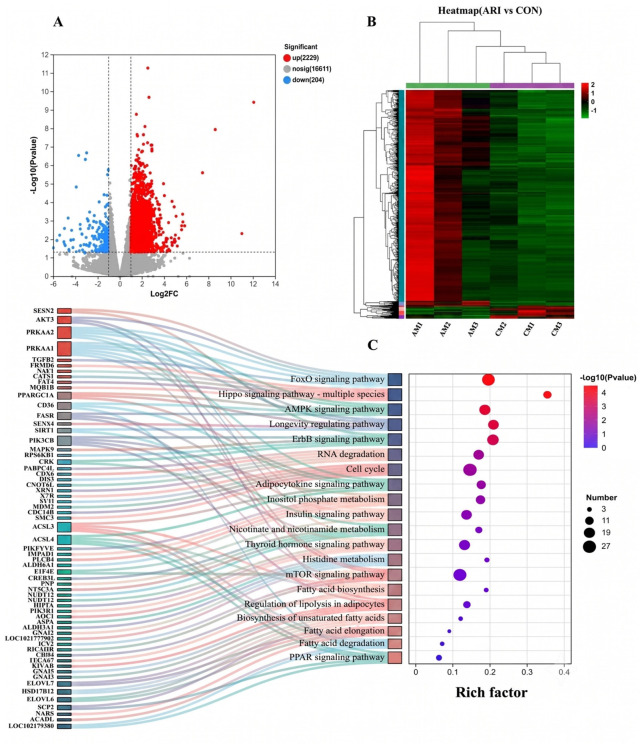
Analysis of DEGs was performed using a volcano plot, cluster analysis of gene sets, and KEGG functional enrichment analysis (ARI vs. CON). CON = Control diet, and ARI = *Artemisia ordosica* Krasch diet. ARI vs. CON: The ARI group compared to the CON group. (**A**) A volcano plot of differentially expressed genes (DEGs) between the ARI and CON groups, depicting significantly upregulated (red), downregulated (blue), and non-significant (gray) genes. (**B**) A hierarchical clustering heatmap of DEGs between the ARI and CON groups, with rows and columns representing individual genes and samples, respectively. The color gradient indicates standardized expression levels, with red representing high expression and blue low expression. (**C**) A Sankey bubble diagram illustrating the KEGG enrichment analysis of DEGs identified in ARI vs. CON. The number of connecting lines and the area of the colored segments for a given gene are proportional to the breadth of its pathway enrichment, implying broader functional involvement.

Transcriptome analysis identified 2433 differentially expressed genes (DEGs) in the ARI group compared to CON, with 2229 genes upregulated and 204 downregulated. Volcano plot (Figure 10A) and hierarchical clustering (Figure 10B) analyses confirmed distinct expression patterns between the groups.

#### 3.8.2. KEGG Pathway Enrichment of DEGs

KEGG enrichment analysis revealed that the key lipid metabolism-related DEGs were significantly enriched in the AMPK signaling pathway (*Q* = 0.0130) and the longevity regulating pathway (*Q* = 0.0125) (Figure 10C and Table 8). In the ARI group, the upregulated genes *PRKAA1*/*2* and *PPARGC1A* were significantly enriched in the AMPK pathway and longevity regulating pathway. The upregulated gene *CD36* and downregulated gene *FASN* were significantly enriched in the AMPK pathways. Concurrently, *ACSL3*/*4* was enriched in the FA biosynthesis and adipocytokine signaling pathway. *ELOVL6*/*7* was enriched in the biosynthesis of unsaturated fatty acids and FA elongation signaling pathways. *SCP2* was enriched in the biosynthesis of unsaturated fatty acids signaling pathway.

### 3.9. Validation of Transcriptome Sequencing by Quantitative Real-Time PCR

**Figure 11:**
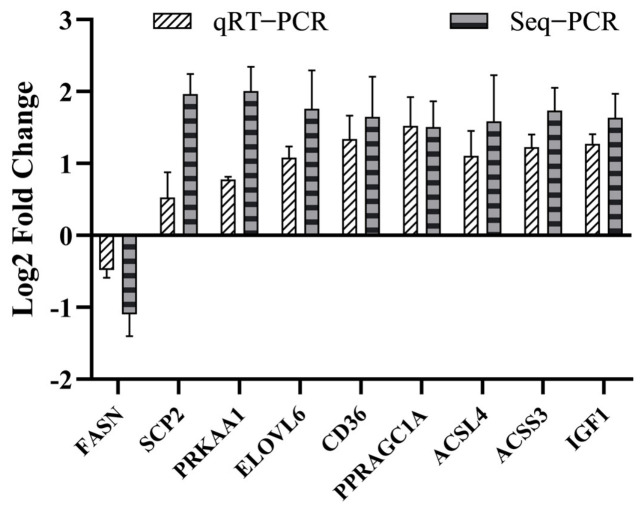
The qPCR validation analysis of differentially expressed genes of RNA-Sequencing (ARI vs. CON). CON = Control diet, and ARI *= Artemisia ordosica* Krasch diet. FC: Fold change in gene expression between the two groups (ARI vs. CON). Log_2_FC: Log_2_-transformed fold change, quantifying the magnitude of differential gene expression under two biological conditions (ARI vs. CON).

The expression patterns of nine chosen DEGs were validated by qRT-PCR, supporting the reproducibility of the RNA-seq results. Relative to the CON group, the expression of *ELOVL6*, *CD36*, *PRKAA1*, *SCP2*, *PPARGC1A*, *ACSL4*, *ACSS3*, and *IGF1* was significantly upregulated (*p* < 0.05) in the ARI group, while *FASN* was significantly downregulated (*p* < 0.05) (Figure 11).

### 3.10. Correlations Between Key Microorganisms, Differential Metabolites, and Phenotypic Parameters

#### 3.10.1. Microbial Correlations with FA Profile and Antioxidant Indices

**Figure 12:**
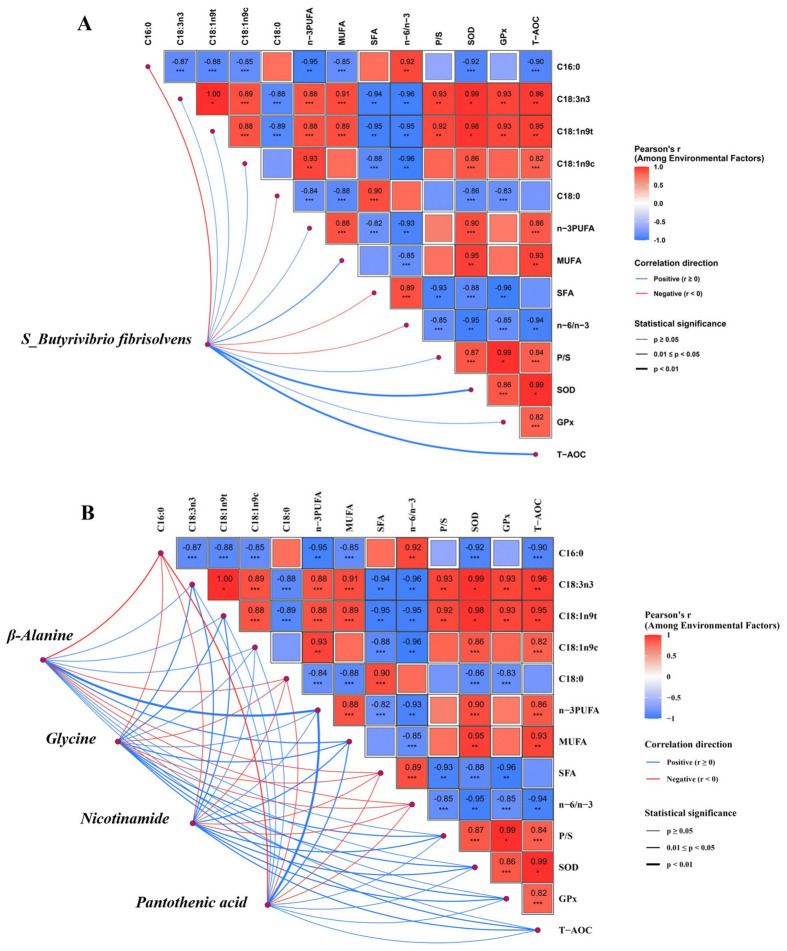
Mantel test heatmaps showing correlations of key differential ruminal bacteria and metabolites with important FA and antioxidant profiles in cashmere goats (ARI vs. CON)**.** CON = Control diet, and ARI = *Artemisia ordosica* Krasch diet. The heatmap illustrates Pearson correlation coefficients among 13 FA derivatives and antioxidant indices. Red for positive correlations, blue for negative ones. (**A**) Network lines depict Mantel test results between *Butyrivibrio fibrisolvens* and the FA/antioxidant profiles. (**B**) Network lines illustrate Mantel test results between four metabolites (β-alanine, glycine, nicotinamide, and pantothenic acid) and the FA/antioxidant profiles. The line color indicates concordant or discordant patterns between overall compositional profiles. Statistical significance is indicated as follows: * *p* < 0.05, ** *p* < 0.01, *** *p* < 0.001.

The relative abundance of *s_Butyrivibrio fibrisolvens* was positively correlated (*p* < 0.05) with pro-atherogenic traits (C16:0, C18:0, total SFA, n-6/n-3 ratio) and negatively correlated (*p* < 0.05) with beneficial FAs (C18:3n3, C18:1n9c, C18:1n9t) and SOD activity (Figure 12A).

#### 3.10.2. Metabolite Correlations with FA Profile and Antioxidant Indices

Metabolites β-alanine, pantothenic acid, and nicotinamide showed a correlation pattern similar to *s_Butyrivibrio fibrisolvens*, positively correlated (*p* < 0.05) with SFA and the n-6/n-3 ratio and negatively correlated with beneficial FAs and SOD activity. In contrast, glycine exhibited an opposite pattern, with a strong positive correlation (*p* < 0.05) with C18:1n9c (Figure 12B).

## 4. Discussion

**Figure 13:**
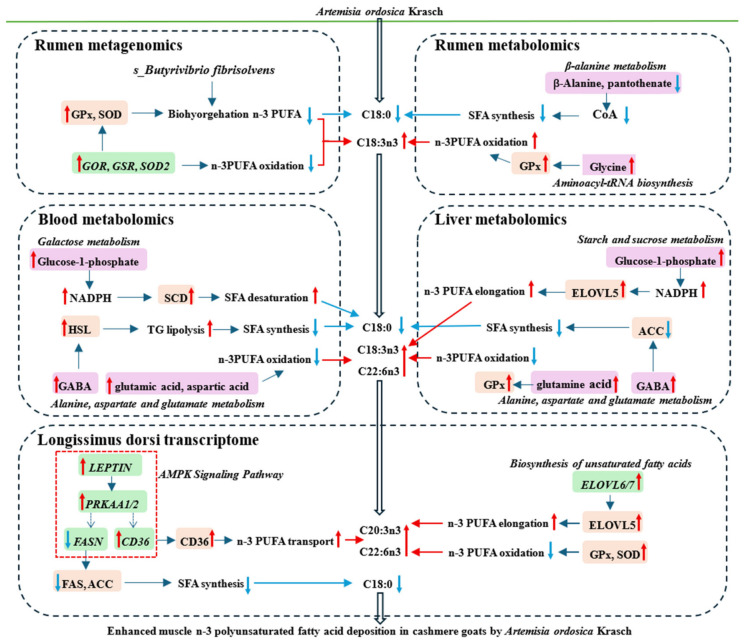
A schematic diagram illustrating the mechanism by which ARI promotes n-3 PUFA deposition in cashmere goat meat. The blue downward arrows denote a significant decrease in the content of the corresponding parameters or significant downregulation of gene expression, whereas the red upward arrows denote a significant increase in content or significant upregulation of gene expression.

The present study found that dietary supplementation with 3% ARI increased the contents of n-3 PUFA including C18:3n3 and C22:6n3, as well as the U/S and t P/S, in the *Longissimus dorsi* of cashmere goats, but reduced the contents of SFAs such as C18:0 and the n-6/n-3. The biohydrogenation of n-3 PUFAs, such as C18:3n3, in the rumen is a primary factor limiting their deposition in ruminant muscle tissue [32]. The water-extracted lyophilized powder of ARI contains polysaccharides, composed of arabinose, galactose, glucose, xylose, and mannose [12]. Our previous study indicated that feeding crude polysaccharides from ARI increased the contents of C18:1n9c, C20:5n3, and the P/S ratio while reducing SFA levels in donkey milk; these effects could be related to the modulation of crude polysaccharides from ARI on the rectal microbiota in donkeys [13]. *S_Butyrivibrio fibrisolvens* is a core ruminal biohydrogenating bacterium [33], and plant-derived active components such as flavonoids and polysaccharides can inhibit its activity, thereby reducing ruminal biohydrogenation and increasing the bypass efficiency of PUFAs [34]. In the present study, rumen metagenomic analysis revealed that ARI supplementation significantly decreased the relative abundance of *s_Butyrivibrio fibrisolvens* in the rumen. The rumen fluid also showed increased levels of biohydrogenation substrates (C18:3n3 and C20:3n3), accumulation of the intermediate product C18:1n9c, and a reduction in the end-product C18:0. These findings suggest that crude polysaccharides in ARI likely inhibit the ruminal biohydrogenation process by reducing the activity of *s_Butyrivibrio fibrisolvens*, thereby decreasing n-3 PUFA hydrogenation and C18:0 production (Figure 13). Furthermore, although dietary nutrient composition and fatty acid profiles did not differ significantly between the CON and ARI diets, the observed alterations in ruminal microbiota and fatty acid profiles may result from ARI bioactive components. However, ARI contains crude polysaccharides as well as other bioactive compounds, including flavonoids and phenolics [11]. Consequently, further studies are warranted to elucidate the specific roles of individual active components in modulating FA metabolism. Meanwhile, we acknowledge that the metagenomic analysis was constrained by a relatively small number of replicates, and rumen biohydrogenation was not directly measured and validated. Given these limitations, future studies with increased biological replicates and direct validation of rumen biohydrogenation are warranted to confirm our findings.

β-Alanine and pantothenate are intermediate and end products of β-alanine metabolism, respectively, with β-alanine serving as a precursor for pantothenate synthesis [35]. Pantothenate is a precursor for coenzyme A (CoA) biosynthesis, and CoA is a core coenzyme that directly activates and regulates FA metabolism, including β-oxidation and de novo SFA synthesis. Thus, reduced CoA synthesis can inhibit SFA production [35]. The present rumen metabolomics analysis demonstrated that ARI significantly decreased the levels of β-alanine and pantothenate, both of which were significantly enriched in the β-alanine metabolism and CoA biosynthesis pathways. This finding partly provides a metabolic explanation for the observed reduction in rumen SFA content in the ARI group (Figure 13).

Glycine, one of the three essential amino acids for glutathione (GSH) synthesis, can enhance GPx activity and overall antioxidant capacity [36]. The aminoacyl-tRNA biosynthesis pathway synthesizes specific aminoacyl-tRNA synthetases with their substrate amino acids, playing an important role in metabolic regulation and alleviating oxidative damage [37]. The current results showed a significant upregulation of glycine content in the ARI group, with significant enrichment in the aminoacyl-tRNA biosynthesis pathway. The *GSR*/*GOR* genes encode glutathione reductase, the functional core of the glutathione metabolic pathway, and their upregulation enhances GPx activity, thereby improving the body’s antioxidant capacity [38]. The present results indicated that ARI increased the relative abundance of microbial *GSR*/*GOR* genes. This implies that ARI may increase the proportion of microbes harboring the *GSR*/*GOR* gene within the community and elevate GPx activity. This speculation is supported by the increase in GPx and T-AOC activities and decreases in MDA observed in the rumen fluid and serum of the ARI group. This reinforced antioxidant microenvironment likely protects oxidation-sensitive n-3 PUFA precursors from peroxidative damage, thereby increasing the content of C18:3n3 and C20:3n3 in rumen fluid and enhancing the flow of n-3 PUFA to the hindgut (Figure 13).

Blood serves as a critical medium for the absorption of n-3 PUFAs from the rumen and their subsequent transport to systemic tissues [39]. Glucose-1-phosphate is a primary product of the galactose metabolism pathway, and elevated levels of glucose-1-phosphate can promote the pentose phosphate pathway to generate NADPH [40], thereby providing essential reducing power for FA desaturation processes [41]. The present serum metabolomics results showed that ARI increased serum glucose-1-phosphate levels, enriched in the galactose metabolism pathway, suggesting that ARI enhanced FA desaturation by modulating galactose metabolism. This is consistent with the observed increase in serum SCD protein abundance and its product, C18:1n9c (Figure 13).

GABA, a regulator of lipid metabolism, promotes triglyceride breakdown, while its direct precursor, glutamate, interconverts with aspartate via transamination, with both serving as glutathione precursors to enhance antioxidant capacity [42]. These three interconnected metabolites are central components of the alanine, aspartate, and glutamate metabolism pathways [43]. Serum and liver metabolomics analysis indicated that ARI significantly upregulated the serum levels of glutamate, aspartate, and GABA, all of which were enriched in the alanine, aspartate, and glutamate metabolism pathways. This suggests that ARI may enhance the production of these metabolites through these pathways, thereby promoting GSH generation, and increasing systemic antioxidant capacity, as supported by elevated GPx and T-AOC activities, and reduced MDA levels in serum and liver. The enhanced systemic antioxidant status likely protects n-3 PUFA and their precursors from oxidative damage during transport to other tissues, ultimately increasing the content of n-3 PUFAs like C20:5n3 and C22:6n3 delivered from blood to peripheral tissues (Figure 13).

In addition, glutamine and GABA are inhibitors of ACC and FAS [44]. The observed decrease in hepatic ACC protein abundance and reduced content of C18:0 in the liver in the ARI group supported this view. Glucose-6-phosphate, a major product of the starch and sucrose metabolism pathway, can drive the pentose phosphate pathway to generate more NADPH, supporting hepatic TCA cycle function and FA transport/elongation processes [45]. CD36 is crucial for free FA uptake [46], and ELOVL5 catalyzes PUFA carbon chain elongation [47]. The present results showed that ARI increased hepatic glucose-6-phosphate levels, significantly enriched in the starch and sucrose metabolism pathway, which may support hepatic TCA cycle energy supply and FA transport/elongation. This aligns with our finding that ARI enhanced hepatic CD36 and ELOVL5 protein abundance. The upregulation of these activities likely promotes hepatic uptake of circulating n-3 PUFA precursors and their subsequent carbon chain elongation, potentially driving the synthesis and accumulation of n-3 LCPUFAs like C22:6n3 in the liver (Figure 13). Hepatic FAs enter the bloodstream and are subsequently transported to peripheral tissues for uptake, utilization, or storage [48]. However, the present study is limited by the lack of functional validation of key metabolites and the small number of replicates. Given these limitations, further investigations with increased replicates and experimental validation are required to elucidate the underlying mechanisms.

The *Longissimus dorsi* is a key site for FA metabolism and deposition [9]. The *PRKAA1* and *PRKAA2* genes encode the catalytic α1 and α2 subunits of AMP-activated protein kinase (AMPK). As an upstream hormonal signal, *LEPTIN* upregulation directly phosphorylates the α2 catalytic subunit of AMPK in muscle, thereby activating the AMPK pathway [49]. AMPK acts as a cellular energy sensor, and its activation (mediated by *PRKAA1*/*2*) promotes lipolysis and downregulates *FASN* expression [50]; this may have restricted de novo SFA synthesis. Concurrently, AMPK activation can upregulate its downstream target gene *PPARGC1A*, which is known to enhance the β-oxidation of SFA [51]. In the present study, *Longissimus dorsi* transcriptome analysis revealed that ARI upregulated the expression of *PRKAA1*, *PRKAA2*, *PPARGC1A*, and *CD36*, while downregulating *FASN* expression, and qRT-PCR results validated these findings in the ARI group. These genes were significantly enriched in the AMPK signaling pathway, suggesting that ARI may activate the AMPK pathway by upregulating *PRKAA1* and *PRKAA2*, leading to increased *PPARGC1A* and *CD36* expression and decreased *FASN* expression. This would promote SFA oxidative breakdown and transport while inhibiting SFA synthesis in muscle, consistent with the observed decrease in FAS protein abundance, increase in CD36 protein abundance, and reduction in C18:0 content in muscle (Figure 13). The replicate numbers for metabolomics and transcriptomics in this study are relatively small, which might render them relatively limited for drawing pathway-level mechanistic conclusions. In addition, the AMPK pathway was not functionally validated, which may further limit the conclusions.

*ACSL3*/*4* enhances the uptake and activation of precursor FAs such as C18:3n3 and C18:1n9c in muscle tissue [52]. *ELOVL6*/*7* functions in elongating long-chain FAs [53]. FA biosynthesis and degradation pathways, as well as unsaturated FA biosynthesis and elongation pathways, are closely associated with n-3 PUFA synthesis [54]. The present transcriptome results showed that ARI upregulated *ACSL3*/*4* and *ELOVL6*/*7* expression, and the qRT-PCR results validated these findings in the ARI group. *ACSL3*/*4* was enriched in FA biosynthesis and degradation pathways, while *ELOVL6*/*7* was enriched in unsaturated FA biosynthesis and elongation pathways. This indicates that ARI may upregulate these genes via the aforementioned metabolic pathways, promoting the activation and carbon chain elongation of precursor FAs like C18:3n3 and C18:1n9c in muscle, this may facilitate the generation of n-3 LCPUFAs such as C22:6n3 within muscle tissue. Furthermore, this anabolic process may be supported by our observations of increased CD36, GPx, and SOD protein abundance and activities in the muscle of the ARI group. Enhanced CD36 protein abundance could facilitate greater uptake of free n-3 PUFAs from blood into muscle for elongation or deposition, while elevated GPx and SOD activities may protect intramuscular n-3 PUFAs from oxidative degradation during synthesis and elongation processes. These combined effects would ultimately increase the content of n-3 PUFAs, including C18:3n3 and C22:6n3, and reduce n-6/n-3 in the *Longissimus dorsi* (Figure 13).

In summary, ARI optimizes the FA profile of cashmere goat meat and enhances the deposition of n-3 LCPUFAs, such as C22:6n-3, which may be associated with coordinated modulation of ruminal biohydrogenation, systemic antioxidant capacity, and intermediary lipid metabolism. However, the relatively small number of replicates in this study limits the robustness of our findings. Thus, further studies incorporating increased replicates and functional validation of key ruminal microbiota, proteins, and metabolites are warranted. Notably, the absence of significant changes in growth performance indicates that the observed alterations in fatty acid profiles and metabolic parameters occurred without compromising overall animal productivity. However, the numerically lower ADG and higher FGR in the 3% ARI group compared with the CON group may indicate a high dosage of ARI. Given that only a single dose (3%) was used in this study, further research with graded doses is required to confirm this possibility.

## 5. Conclusions

The deposition of n-3 PUFAs such as C18:3n3 and C22:6n3 in the muscle of cashmere goats is promoted by ARI supplementation, and the content of SFAs including C18:0 showed the decreases. These improvements were associated with a reduced relative abundance of the core biohydrogenating bacterium *s_Butyrivibrio_fibrisolvens*, and enhanced systemic antioxidant capacity and upregulation of the AMPK pathway-related genes involved in lipid metabolism (Figure 13). Collectively, these findings suggest that ARI may serve as a promising functional feed ingredient for producing healthier cashmere goat meat with an improved nutritional FA profile. Nevertheless, the mechanisms proposed herein are primarily based on associative multi-omics evidence, and the specific contributions of ARI-derived bioactive compounds remain to be elucidated. Future studies employing purified ARI components and targeted functional validation of the candidate microbes, metabolites, and genes identified in this study are warranted to establish causal relationships underlying ARI-mediated regulation of n-3 PUFA deposition.

## Figures and Tables

**Table 1:** Composition and analysis of experimental diets fed to cashmere goats.

Item	Days 1 to 30		Days 31 to 60		Days 61 to 90	
	CON ^4^	ARI ^5^	CON	ARI	CON	ARI
Ingredients (% air dry basis)						
Alfalfa hay	25.60	24.09	14.60	13.71	12.18	11.44
Corn stalk	5.07	4.77	19.67	18.47	24.60	23.10
Oat grass	20.22	19.03	14.79	13.88	12.33	11.57
*Artemisia ordosica* Krasch ^1^	0.00	3.00	0.00	3.00	0.00	3.00
Corn	27.46	27.46	31.76	31.76	32.30	32.30
Soybean meal	11.44	11.44	9.69	9.69	8.16	8.16
Linseed cake	5.11	5.11	3.28	3.28	4.21	4.21
DDGS	2.91	2.91	4.11	4.11	4.12	4.12
Premix ^2^	0.52	0.52	0.48	0.48	0.48	0.48
Limestone	0.21	0.21	0.19	0.19	0.19	0.19
CaHPO_4_	0.21	0.21	0.19	0.19	0.19	0.19
NaCl	0.57	0.57	0.48	0.48	0.48	0.48
NaHCO_3_	0.37	0.37	0.76	0.76	0.76	0.76
Magnesia	0.31	0.31	0.00	0.00	0.00	0.00
Total	100.00	100.00	100.00	100.00	100.00	100.00
Nutrition level						
Digestible energy ^3^ (MJ/kg)	12.83	12.81	12.87	12.73	12.73	12.70
Crude protein	18.87	18.66	16.28	16.28	15.35	15.32
Ether extract	2.91	3.12	2.90	3.10	2.68	2.87
Neutral detergent fiber	42.53	42.05	44.86	44.31	45.74	45.46
Acid detergent fiber	23.22	23.01	24.26	23.88	24.77	24.77
Calcium	1.13	1.14	1.05	1.08	1.03	1.05
Phosphorous	0.46	0.46	0.45	0.45	0.43	0.43

^1^ The crude protein content of *Artemisia ordosica* Krasch (dry matter basis %) is 11.04%, the ether extract content is 7.74%, the crude fiber content is 29.39%, the nitrogen-free extract content is 35.25%, the ash content is 9.68%, the calcium content is 3.45%, and the phosphorus content is 0.54%. ^2^ Provided per kg of premix: vitamin A 1.2 × 10^6^ IU, D_3_ 5 × 10^5^ IU, E 5 × 10^3^ IU, K_3_ 360 mg, B_1_ 70 mg, B_2_ 1.7 g, B_6_ 180 mg, nicotinic acid 4.4 g, D-pantothenic acid 3.4 g, B_12_ 6 mg, biotin 28 mg, folic acid 300 mg, Fe 8 g, Cu 1.6 g, Zn 10 g, Mn 6 g, I 60 mg, Se 60 mg, Co 50 mg. ^3^ Digestible energy was derived by calculation, whereas the remaining parameters were experimentally determined. ^4^ CON = Control diet. ^5^ ARI = *Artemisia ordosica* Krasch diet.

**Table 2:** Effects of ARI on growth performance of cashmere goats.

Item	CON ^1^	ARI ^2^	SEM	*p-*Value
Initial BW, kg	18.10	18.36	0.575	0.760
Final BW, kg	25.82	23.65	0.966	0.143
ADG g/d	94.66	73.33	6.375	0.144
DMI, g/d	850.00	760.00	5.362	0.246
FGR	9.71	12.05	1.108	0.135

^1^ CON = Control diet. ^2^ ARI = *Artemisia ordosica* Krasch diet.

**Table 3:** Effects of ARI on FA profiles (percentage of total identified FAs methyl esters) in rumen fluid and plasma of cashmere goats.

Item	Rumen Fluid				Plasma			
	CON ^1^	ARI ^2^	SEM	*p*-Value	CON	ARI	SEM	*p*-Value
C10:0	1.78	1.58	0.100	0.067	1.57	2.09	0.219	0.070
C12:0	2.17	2.00	0.116	0.163	1.56	1.65	0.163	0.527
C13:0	0.98	0.82	0.047	0.005	0.78	0.89	0.133	0.261
C14:0	3.70	3.48	0.336	0.525	2.36	2.71	0.212	0.126
C15:0	2.13	2.11	0.242	0.915	1.27	1.48	0.157	0.089
C16:0	14.52	12.96	0.524	0.010	14.49	13.70	0.767	0.122
C17:0	2.11	1.75	0.189	0.184	2.03	2.16	0.131	0.361
C18:0	13.76	12.07	0.784	0.049	19.92	17.80	1.932	0.021
C20:0	2.43	2.21	0.133	0.114	1.74	1.94	0.178	0.385
C21:0	0.94	0.85	0.048	0.077	0.98	1.31	0.141	0.017
C22:0	2.10	1.92	0.158	0.108	2.37	1.67	0.216	0.014
C14:1	1.64	1.57	0.145	0.654	1.04	1.19	0.134	0.278
C15:1	0.71	0.73	0.080	0.784	0.89	1.16	0.151	0.122
C16:1	1.86	1.89	0.245	0.894	1.38	1.70	0.128	0.017
C17:1	1.02	1.89	0.853	0.344	1.09	1.46	0.092	0.033
C18:1n9t	8.32	10.83	1.167	0.048	1.51	2.05	0.285	0.010
C18:1n9c	11.00	12.89	0.753	0.025	17.30	18.99	1.36	0.351
C20:1	1.00	0.90	0.037	0.017	0.94	0.84	0.097	0.293
C24:1	0.97	0.91	0.040	0.250	0.77	0.91	0.131	0.495
C18:2n6t	0.90	0.81	0.035	0.192	0.95	1.31	0.169	0.024
C18:2n6c	6.23	6.54	1.115	0.786	12.75	10.88	0.229	0.069
C18:3n6	0.92	0.84	0.036	0.255	1.09	1.33	0.131	0.108
C20:2n6	0.92	0.87	0.042	0.201	0.90	1.28	0.095	0.008
C20:3n6	0.84	0.80	0.172	0.761	0.88	1.31	0.077	0.002
C20:4n6	0.81	0.75	0.165	0.739	2.45	2.24	0.148	0.248
C22:2n6	0.93	0.87	0.034	0.152	0.83	0.90	0.078	0.048
C18:3n3	1.90	2.52	0.174	0.003	2.23	2.80	0.206	0.048
C20:3n3	0.87	1.02	0.177	0.026	0.95	1.24	0.079	0.017
C20:5n3	0.91	0.86	0.181	0.252	1.30	1.91	0.155	0.001
C22:6n3	0.97	1.01	0.052	0.552	1.18	1.83	0.120	0.010
SFA	54.24	48.92	2.138	0.027	49.59	48.62	1.323	0.375
UFA	45.76	51.08	2.138	0.027	50.41	51.38	1.323	0.375
MUFA	28.63	34.1	2.137	0.025	27.63	27.54	1.105	0.652
PUFA	16.45	16.87	1.009	0.681	26.45	28.68	1.117	0.016
n-3 PUFA	4.68	5.36	0.260	0.026	5.59	7.85	0.877	0.001
n-6 PUFA	11.77	11.52	0.995	0.804	21.00	20.53	2.082	0.380
n-3 LCPUFA	2.78	2.84	0.176	0.770	3.36	5.05	0.861	0.001
n-6 LCPUFA	3.71	3.25	0.190	0.029	6.75	7.00	0.778	0.599
n-6/n-3	2.55	2.09	0.224	0.031	3.89	2.65	0.344	0.001
U/S	0.85	1.06	0.095	0.049	1.02	1.06	0.205	0.401
P/S	0.31	0.35	0.026	0.024	0.53	0.60	0.115	0.019

^1^ CON = Control diet. ^2^ ARI = *Artemisia ordosica* Krasch diet.

**Table 4:** Effects of ARI on FA profiles (percentage of total identified FAs methyl esters) in liver and *Longissimus dorsi* of cashmere goats.

Item	Liver				*Longissimus dorsi*			
	CON ^1^	ARI ^2^	SEM	*p*-Value	CON	ARI	SEM	*p*-Value
C10:0	0.41	0.51	0.226	0.108	0.87	0.86	0.250	0.892
C12:0	0.57	0.59	0.295	0.934	0.90	0.83	0.191	0.428
C13:0	0.48	0.49	0.109	0.866	0.53	0.64	0.198	0.265
C14:0	1.29	1.26	0.245	0.908	2.58	1.93	0.341	0.088
C15:0	0.78	0.66	0.138	0.390	0.69	0.66	0.142	0.675
C16:0	15.70	15.36	1.266	0.790	15.78	15.40	2.486	0.698
C17:0	2.00	1.88	0.218	0.579	6.05	5.29	4.415	0.197
C18:0	25.57	20.31	2.094	0.027	16.34	11.69	1.894	0.012
C20:0	0.90	0.31	0.231	0.030	1.45	1.23	0.344	0.034
C21:0	0.91	1.04	0.184	0.497	0.81	0.76	0.170	0.457
C22:0	1.04	0.54	0.251	0.048	0.99	0.91	0.305	0.191
C14:1	0.56	0.61	0.117	0.646	0.64	0.57	0.194	0.735
C15:1	0.35	0.32	0.108	0.803	0.57	0.49	0.146	0.869
C16:1	1.06	1.17	0.188	0.551	1.52	1.69	0.088	0.094
C17:1	0.89	0.87	0.120	0.838	1.49	1.30	0.176	0.207
C18:1n9t	0.86	0.89	0.170	0.620	0.86	1.08	0.180	0.001
C18:1n9c	16.20	22.07	4.935	0.036	32.00	33.11	3.039	0.721
C20:1	0.46	0.39	0.138	0.643	0.67	0.54	0.035	0.003
C24:1	0.65	0.63	0.230	0.938	0.59	0.61	0.167	0.716
C18:2n6t	0.47	0.40	0.160	0.668	0.59	0.74	0.196	0.227
C18:2n6c	11.32	11.93	1.158	0.607	4.10	3.83	0.549	0.630
C18:3n6	0.64	0.57	0.127	0.573	0.59	0.59	0.072	0.965
C20:2n6	0.52	0.38	0.163	0.417	0.71	0.71	0.241	0.982
C20:3n6	1.13	0.86	0.113	0.030	0.88	0.70	0.262	0.191
C20:4n6	12.77	13.31	1.570	0.758	2.69	2.57	0.484	0.648
C22:2n6	0.52	0.42	0.142	0.417	0.80	0.91	0.050	0.037
C18:3n3	1.44	1.52	0.094	0.034	0.97	1.35	0.186	0.001
C20:3n3	0.47	0.57	0.118	0.407	0.71	0.83	0.230	0.346
C20:5n3	2.85	3.03	0.332	0.598	1.28	1.12	0.256	0.131
C22:6n3	2.66	3.18	0.388	0.024	0.91	1.29	0.260	0.029
SFA	49.36	43.70	2.343	0.041	47.52	40.81	4.074	0.007
UFA	50.64	56.30	2.343	0.041	52.48	59.19	2.840	0.007
MUFA	15.76	21.58	6.106	0.037	38.95	41.45	4.855	0.277
PUFA	34.73	35.41	2.835	0.816	14.07	14.65	1.563	0.471
n-3 PUFA	7.27	8.41	0.393	0.015	3.92	4.36	0.695	0.036
n-6 PUFA	27.43	25.04	3.223	0.475	11.28	10.21	2.157	0.010
n-3 LCPUFA	5.76	6.96	0.404	0.010	2.82	3.09	0.411	0.047
n-6 LCPUFA	15.00	14.54	1.494	0.761	5.09	4.90	1.123	0.656
n-6/n-3	3.37	3.07	0.219	0.197	2.65	2.27	0.276	0.019
U/S	1.05	1.30	0.106	0.037	1.12	1.48	0.179	0.005
P/S	0.68	0.80	0.050	0.043	0.29	0.37	0.100	0.012

^1^ CON = Control diet. ^2^ ARI = *Artemisia ordosica* Krasch diet.

**Table 5:** Summary of significant rumen *Butyrivibrio*-related bacteria from LEfSe LDA (CON vs. ARI) ^1^.

Species Name	Group	Mean	LDA ^3^	*p*-Value
*s__Butyrivibrio_sp* ^2^ *__XBB1001*	CON	2.7711	2.1461	0.0495
*s__Butyrivibrio_sp__AE2015*	CON	2.8950	2.2155	0.0495
*s__Butyrivibrio_sp__AC2005*	CON	2.9921	2.1310	0.0495
*s__Butyrivibrio_sp__LC3010*	CON	2.7673	2.0429	0.0495
*s__Butyrivibrio_sp__AE3004*	CON	2.8906	2.2496	0.0495
*s__Butyrivibrio_sp__FC2001*	CON	2.7533	2.2090	0.0495
*s__Butyrivibrio_fibrisolvens*	CON	3.5546	2.8795	0.0495

^1^ CON vs. ARI: The relative abundance of the following bacterial taxa was significantly higher in the CON group compared to the ARI group. CON = Control diet; ARI = *Artemisia ordosica* Krasch diet. ^2^ Sp is an abbreviation for a species that remains unclassified and lacks a formal taxonomic name. ^3^ The linear discriminant analysis (LDA) score indicates a greater likelihood of a taxon serving as a discriminative biomarker, with a conventional significance threshold set at LDA > 2.0.

**Table 6:** Key lipid metabolism pathways and differential abundance genes ^1^ identified in the ARI Group via KEGG functional enrichment analysis of the Rumen Microbial Gene Catalog (ARI vs. CON) ^2^.

Pathway ID	Description	Significance ^3^	UP_GENE	Down_Gene
ko04152	AMPK signaling pathway	yes	*PRKAA*, *AMPK*, *LIPE*, *HSL*	*PFKA*, *PFK*, *FBP*
ko04910	Insulin signaling pathway	yes	*PRKAA*, *AMPK*, *LIPE*, *HSL*	*PYG*, *GLGP*, *FBP*
ko04211	Longevity regulating pathway	yes	*PRKAA*, *AMPK*	*SESN2*
ko04918	Thyroid hormone synthesis	yes	*GSR*, *GOR*	*ALB*
ko04920	Adipocytokine signaling pathway	yes	*PRKAA*, *AMPK*, *ACSL*, *FADD*	*ACSBG*
ko04923	Regulation of lipolysis in adipocytes	yes	*LIPE*, *HSL*	*MGLL*
ko04068	FoxO signaling pathway	yes	*SOD2*	*ATM*
ko00480	Glutathione metabolism	ns	*GSS*, *GSHB*, *GSHA*, *GSR*, *GOR*, *GST*	*PGD*, *GND*, *GNTZ*

^1^ Differential abundance genes (DAGs), in contrast to differentially expressed genes (DEGs), are defined by comparative analysis of gene occurrence frequency (abundance) between groups, reflecting differences in the relative abundance of specific microbial genes. ^2^ ARI vs. CON: Analysis revealed differential expression of the following genes in the ARI group relative to the CON group. CON = Control diet; ARI = *Artemisia ordosica* Krasch diet. ^3^ Significance (enrichment status): Functional pathways were considered significantly enriched (yes) when the absolute value of the ReporterScore was ≥1.65; otherwise, they were considered not significant (ns).

**Table 7:** Lipid metabolism-related pathways enriched for differential metabolites in ruminal fluid, serum and liver (ARI vs. CON) ^1^.

Pathway ID	Pathway Name	*Q*-Value ^2^	Upregulated Metabolites	Downregulated Metabolites
Ruminal Fluid				
ko00970	Aminoacyl-tRNA biosynthesis	0.0000	glycine; methionine	glutamic acid; lysine;
ko00410	beta-Alanine metabolism	0.0088		beta-Alanine;4-aminobutyric acid; pantothenic acid;1,3-diaminopropane
ko04212	Longevity regulating pathway-worm	0.0166	oleic acid	nicotinamide
ko00770	Pantothenate and CoA biosynthesis	0.0427		beta-Alanine; pantothenic acid
Serum				
ko00250	Alanine, aspartate and glutamate metabolism	0.0000	glutamic acid; aspartic acid; asparagine; 4-aminobutyric acid; N-acetyl-L-aspartic acid	
ko00970	Aminoacyl-tRNA biosynthesis	0.0000	glutamic acid; aspartic acid; proline; asparagine	
ko00410	beta-Alanine metabolism	0.0004	aspartic acid; 4-aminobutyric acid	malonic acid
ko00052	Galactose metabolism	0.0074	Glucose-1-phosphate; myo-inositol	Galactinol
Liver				
ko00970	Aminoacyl-tRNA biosynthesis	0.0000	glutamine	glycine; lysine; tyrosine; leucine; histidine; asparagine; Isoleucine
ko00250	Alanine, aspartate and glutamate metabolism	0.0111	glutamine; fumaric acid; 4-aminobutyric acid	asparagine
ko00500	Starch and sucrose metabolism	0.0111	Glucose-1-phosphate; trehalose-6-phosphate; trehalose	
ko00910	Nitrogen metabolism	0.0112	glutamine	hydroxylamine
ko04212	Longevity regulating pathway—worm	0.0165	nicotinamide; oleic acid	

^1^ ARI vs. CON: Comparative analysis revealed significantly altered levels of the following metabolites in the ARI group relative to the CON group. CON = Control diet; ARI = *Artemisia ordosica* Krasch diet. ^2^ *Q*-value: false discovery rate (FDR)-adjusted *p*-value derived from the Benjamini–Hochberg multiple testing correction. *Q*-value < 0.05 was set as the significance threshold.

**Table 8:** Important KEGG pathways related to lipid metabolism enriched in the *Longissimus dorsi* (ARI vs. CON) ^1^.

Pathway ID	Pathway Name	Q-Value ^2^	Upregulated Gene	Downregulated Gene
map04152	AMPK signaling pathway	0.0130	*AKT3*, *CREB3L1*, *PRKAA2*, *PRKAA1*, *CD36*, *PPARGC1A*, *LEPTIN*, *IGF1*, *PIK3CA*	*CREB3L1*, *PFKFB3*, *FASN*, *HNF4A*, *PFKL*
map04211	Longevity regulating pathway	0.0125	*EIF4E*, *SESN3*, *SIRT1*, *IGF1*, *PRKAA2*, *PIK3R1*, *AKT3*, *PRKAA1*, *PIK3CB*, *PPARGC1A*, *PRKACB*, *PIK3CA*	*LOC102188072*, *LOC102178383*, *LOC108633298*, *LOC102188618*
map04920	Adipocytokine signaling pathway	0.1009	*ACSL3*, *MAPK8*, *LEPTIN*, *ACSL4*, *CD36*, *AKT3*, *JAK2*, *CHUK*, *PRKAA1*, *MAPK9*, *PPARGC1A*, *PRKAA2*	*LOC106504022*
map00061	FA biosynthesis	0.3936	*CBR4*, *ACSL4*, *ACSL3*	*FASN*
map01040	Biosynthesis of unsaturated fatty acids	0.7509	*ELOVL6*, *SCP2*, *ELOVL7*	
map00062	FA elongation	0.9850	*ELOVL6*, *ELOVL7*	

^1^ ARI vs. CON: Comparative analysis revealed significantly altered levels of the following metabolites in the ARI group relative to the CON group. CON = Control diet; ARI = *Artemisia ordosica* Krasch diet. ^2^ *Q*-value: false discovery rate (FDR)-adjusted *p*-value derived from the Benjamini–Hochberg multiple testing correction. *Q*-value < 0.05 was set as the significance threshold.

## Data Availability

The raw transcriptomic and metagenomic sequencing data have been deposited in the NCBI BioProject (PRJNA1440623) and CNSA (CNPO009720), respectively.

## Abbreviations

The following abbreviations are used in this manuscript:

ARI	*Artemisia ordosica* Krasch
ACC	Acetyl-CoA carboxylase
CD36	Cluster of differentiation 36
ELOVL	Elongation of very long-chain fatty acids protein
FAS	Fatty acid synthase
HSL	Hormone-sensitive lipase
SCD	Stearoyl-CoA desaturase
TG	Triglycerides
NEFA	Non-esterified fatty acids
GLU	Glucose
SOD	Superoxide dismutase
T-AOC	Total antioxidant capacity
MDA	Malondialdehyde
GPx	Glutathione peroxidase
FA	Fatty acid
SFA	Saturated fatty acid
LCPUFA	Long-chain polyunsaturated fatty acid
MUFA	Monounsaturated fatty acid
PUFA	Polyunsaturated fatty acid
UFA	Unsaturated fatty acid
DAGs	Differential abundance genes
DEGs	Differentially expressed genes
HMDB	Human Metabolome Database
ACSL	Acyl-CoA synthetase long chain family member
ACSS3	Acyl-CoA synthetase short chain family member 3
LIPE	Lipase E, hormone-sensitive type
AMPK	AMP-activated protein kinase
GABA	γ-aminobutyric acid
PPARGC1A	PPARG coactivator 1 alpha
PRKAA	Protein kinase AMP-activated catalytic subunit alpha
PGD	Phosphogluconate dehydrogenase
NADPH	Nicotinamide adenine dinucleotide phosphate
SCP2	Sterol carrier protein 2

## Appendix A

**Table A1:** Dietary FA profiles (percentage of total identified FA methyl esters).

Item	Days 1 to 30		Days 31 to 60		Days 61 to 90	
	CON ^1^	ARI ^2^	CON	ARI	CON	ARI
C10:0	0.76	0.91	0.83	0.97	0.82	0.96
C12:0	0.90	0.91	0.94	0.94	0.93	0.93
C13:0	0.25	0.25	0.25	0.25	0.25	0.25
C14:0	1.16	1.18	1.17	1.18	1.16	1.16
C15:0	0.62	0.62	0.62	0.62	0.61	0.61
C16:0	16.35	16.12	16.26	16.13	15.81	15.72
C17:0	0.78	0.78	0.80	0.80	0.80	0.80
C18:0	3.06	3.04	3.05	3.02	3.01	2.99
C20:0	1.29	1.32	1.30	1.33	1.29	1.32
C21:0	0.39	0.40	0.40	0.40	0.39	0.39
C22:0	1.29	1.32	1.30	1.33	1.29	1.32
C14:1	0.33	0.34	0.36	0.37	0.36	0.37
C15:1	0.16	0.15	0.13	0.12	0.12	0.11
C16:1	0.65	0.66	0.70	0.70	0.70	0.70
C17:1	0.58	0.59	0.62	0.62	0.62	0.62
C18:1n9t	0.47	0.49	0.46	0.47	0.44	0.45
C18:1n9c	13.51	13.66	15.39	15.38	15.78	15.77
C20:1	0.71	0.72	0.75	0.75	0.76	0.76
C22:1	0.40	0.39	0.40	0.38	0.42	0.40
C18:3n3	17.16	16.57	13.07	12.85	13.16	13.00
C20:3n3	1.05	1.05	1.05	1.05	1.04	1.04
C20:5n3	0.73	0.74	0.77	0.77	0.77	0.77
C22:6n3	0.76	0.78	0.91	0.92	0.95	0.96
C18:2n6c	31.68	31.75	34.06	34.06	34.18	34.18
C18:3n6	0.49	0.49	0.38	0.38	0.34	0.35
C20:2n6	0.54	0.55	0.55	0.55	0.55	0.55
C20:3n6	0.35	0.34	0.26	0.24	0.23	0.22
C20:4n6	0.54	0.55	0.49	0.50	0.46	0.47
C22:2n6	0.52	0.51	0.41	0.39	0.37	0.35
SFA	28.27	28.58	28.17	28.41	27.70	27.87
UFA	71.73	71.42	71.83	71.59	72.30	72.13
MUFA	17.35	17.54	19.37	19.36	19.77	19.75
PUFA	54.38	53.88	52.46	52.23	52.53	52.37

^1^ CON = Control diet. ^2^ ARI = *Artemisia ordosica* Krasch diet.

**Table A2:** Primer pairs sequences for quantitative real-time PCR.

Gene	Primer Sequences (5′-3′)	GenBank Accession Number	Annealing Temperature (°C)	Length (bp)
*B2M*	F ^1^: ggtgctgcttagaggtctcg	NM_001009284	59	109
	R ^2^: acgctgagttcactcccaac			
*FASN*	F: gcaaccaggggagaccgt	AB011671	60	300
	R: ctgagggcaatggcgatgg			
*PRKAA1*	F: accctcccatttgatgatga	NM001112816	58	97
	R: tggcaacagaacgattgaga			
*SCP2*	F: acgattgcttttctgccaat	NM_001033990	60	71
	R: tccaccttgaccttctggac			
*ELOVL6*	F: ctctggtctctgacccttgc	XM_004009618	57	89
	R: aggcctttggtcatcacagt			
*CD36*	F:ccatctttgaaccctcgc	NM_001285578.1	54	196
	R:ggatccgtatagccccac			
*PPARGC1A*	F:tgtgcaaccaggactctgta	NM_001285634.1	60	148
	R:ttgagtccacccagaaagctg			
*ACSL4*	F:tgctgggagggaatgtccgtat	XM_018044177.1	61	227
	R:accgccttcctgccagtctt			
*ACSS3*	F:gacttaggctgggttgttgga	XM_013963698.2	57	128
	R:gcgagcacacggaaatagg			
*IGF1*	F:aaaatcagcagtcttccaacccaa	NM_001285697.1	60	124
	R:tgaaggcgagcaagcacagg			

^1^ F = Forward primer. ^2^ R = Reverse primer.
